# Radiomics in pulmonary neuroendocrine tumours (NETs)

**DOI:** 10.1007/s11547-022-01494-5

**Published:** 2022-05-10

**Authors:** Diletta Cozzi, Eleonora Bicci, Edoardo Cavigli, Ginevra Danti, Silvia Bettarini, Paolo Tortoli, Lorenzo Nicola Mazzoni, Simone Busoni, Silvia Pradella, Vittorio Miele

**Affiliations:** 1 Department of Emergency Radiology, Careggi University Hospital, Largo Brambilla 3, 50134 Florence, Italy; 2 Italian Society of Medical and Interventional Radiology (SIRM), SIRM Foundation, 20122 Milan, Italy; 3 Department of Health Physics, Careggi University Hospital, Largo Brambilla 3, 50134 Florence, Italy; 4 Department of Health Physics, AUSL Toscana Centro, Via Ciliegiole 97, 51100 Pistoia, Italy

**Keywords:** Lung carcinoids, Computed tomography, Radiomics, Ki-67

## Abstract

**Objectives:**

The aim of this single-centre, observational, retrospective study is to find a correlation using Radiomics between the analysis of CT texture features of primary lesion of neuroendocrine (NET) lung cancer subtypes (typical and atypical carcinoids, large and small cell neuroendocrine carcinoma), Ki-67 index and the presence of lymph nodal mediastinal metastases.

**Methods:**

Twenty-seven patients (11 males and 16 females, aged between 48 and 81 years old—average age of 70,4 years) with histological diagnosis of pulmonary NET with known Ki-67 status and metastases who have performed pre-treatment CT in our department were included. All examinations were performed with the same CT scan (Sensation 16-slice, Siemens). The study protocol was a baseline scan followed by 70 s delay acquisition after administration of intravenous contrast medium. After segmentation of primary lesions, quantitative texture parameters of first and higher orders were extracted. Statistics nonparametric tests and linear correlation tests were conducted to evaluate the relationship between different textural characteristics and tumour subtypes.

**Results:**

Statistically significant (*p* < 0.05) differences were seen in post-contrast enhanced CT in multiple first and higher-order extracted parameters regarding the correlation with classes of Ki-67 index values. Statistical analysis for direct acquisitions was not significant. Concerning the correlation with the presence of metastases, one histogram feature (Skewness) and one feature included in the Gray-Level Co-occurrence Matrix (ClusterShade) were significant on contrast-enhanced CT only.

**Conclusions:**

CT texture analysis may be used as a valid tool for predicting the subtype of lung NET and its aggressiveness.

## Introduction

Pulmonary neuroendocrine tumours (NETs) are a heterogeneous group of malignant neuroendocrine diseases accounting for approximately 25% of all NETs and 2% of all lung malignancies [1, 2]. Pulmonary NETs arise from Kulchitzky cells of the bronchial mucosa and are differentiated into four groups in increasing order of aggressiveness in low grade (typical carcinoids, TCs), intermediate grade (atypical carcinoids, ACs), high grade (large-cell neuroendocrine carcinomas, LCNECs or small-cell lung carcinomas, SCLC) [3–5]. These different tumour forms show progressively increasing numbers of mitoses, with the lowest values in typical carcinoids and the highest in SCLC, which is, indeed, the most aggressive form. Carcinoids are divided into typical and atypical, based on the number of mitoses and the presence of necrosis; in particular, typical carcinoids are larger than 0.5 cm showing less than 2 mitoses/2mm^2^ and absence of necrosis, whereas atypical carcinoids show 2 to 10 mitoses/2mm^2^ and/or foci of necrosis [2, 3]. TCs accounts for only 1–2% of primary lung tumours, representing 80–90% of all pulmonary carcinoids with a frequency of 1.4 to 2 per 100,000 people [6, 7]. Their occurrence is not related to cigarette smoking and there are no gender distinctions; only high-grade NETs have a high association with smoking status and metastases occur in 15% of cases [8–11]. Increasing importance is being given to the assay of a nuclear antigen expressed by proliferating cells, Ki-67. This is used in 2019 WHO classification of gastrointestinal neuroendocrine tumours (GEP-NENs), specifically well-differentiated NENs are further divided into grades just based on Ki-67 proliferation index and mitotic index into grade 1 (G1, mitotic rate < 2, Ki-67 index < 3), grade 2 (G2, mitotic rate 2–20, Ki-67 index 3–20), and grade 3 (G3, mitotic rate > 20, Ki-67 index > 20) [12, 13]. Although the use of Ki-67 in staging lung NETs is not yet part of the current grading system, recent studies suggest that this index may play a role in predicting prognosis and in separating TCs from ACs tumours or in differentiating the high-grade SCLS and LCNEC from carcinoid tumours [3, 14, 15]. Chest computed tomography (CT) imaging is the most useful diagnostic tool, because of its high resolution and the ability in evaluating tumour extension, localization, presence of local or distant metastases. NETs appear as lobulated lesions, with irregular margins and eccentric calcifications may be present. The lesions develop predominantly into lung parenchyma, sometimes into a bronchus, or mixed with a dominant extraluminal component with a very small endoluminal portion. In the case of mass-effect obstruction of a bronchus, atelectasis is associated [16]. Carcinoids, after administration of contrast medium, tend to have an intense enhancement, often homogeneous [17]. Because of this overlap of radiological features between the various forms of NETs, the distinction is very difficult. However, differentiating these forms is crucial for staging, prognosis, and treatment. Texture analysis is an innovative technique used to measure tissue heterogeneity based on the distribution of pixel values, not affected by human vision and subjective analysis [18]. Recent studies have shown how the analysis of CT-texture features of the lesions can add information about the tumour that could not be assessed by CT examination alone [18, 19]. Radiomics is a promising method in tumour assessment for predictors of prognosis and treatment response in the oncology field [19–24]. Therefore, this study aims to find a correlation between pulmonary NETs radiomics features and their aggressiveness, expressed as Ki-67 index-rate and the presence of lymph nodal mediastinal metastases.

## Materials and methods

### Patients’ selection

**Fig. 1:**
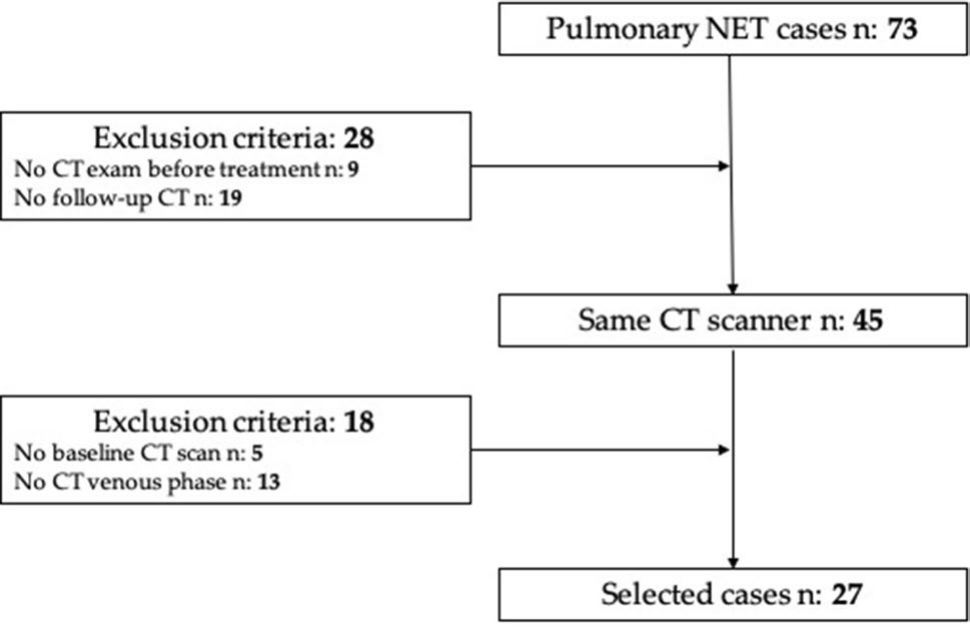
Workflow of patients’ selection

This is a single-centre, observational, retrospective study. Between September 2008 and January 2020, all patients with a histological diagnosis of pulmonary NET were retrospectively analysed. Inclusion criteria were patients aged 18–99 years, histological diagnosis of pulmonary NET (confirmed through biopsy), Ki-67 value, same CT scanner for the baseline examination, at least one follow-up CT or FGD-PET/CT. The initial population included 73 patients; of these 28 did not have a pre-treatment or follow-up CT performed in our department. To obtain a sample as homogeneous as possible, the largest population that performed CT examination with the same protocol in the same CT scanner was considered, resulting in a population of 45 patients. Of these, five did not have a baseline CT scan without contrast medium and 13 did not perform a venous phase acquisition, resulting in a population of 27 patients, 11 males and 16 females (Fig. 1). As there is still no standardization in the use and class division of lung NETs using the Ki-67 index, we relied in our study on the most recent WHO 2019 GEP NENs grading [12]. Selected patients were then divided in three groups: Ki-67 < 3 (Group 1), 3 < Ki-67 < 20 (Group 2), and Ki-67 > 20 (Group 3).

### Images acquisitions and analysis

All examinations were performed with the same CT scan (Sensation 16-slice, Siemens). The study protocol was a baseline scan followed by 70 s delay acquisition after administration of intravenous contrast medium (flow rate 3 mL/s, followed by a 40-ml bolus of saline solution, calculated on total body weight). The acquisition parameters for unenhanced scans were matrix size 512 × 512 pixels with slice thickness between 1 and 5 mm, 120 kVp, 145 ± 97 mAs, B10f, B20f or B30f convolution kernels, CTDIvol of 10.3 ± 6.7 mGy, DLP in the range 237/1226 mGy*cm; for contrast-enhanced scans acquisition parameters were matrix size 512 × 512 pixels with slice thickness between 1 and 5 mm, 120 kVp, 156 ± 101 mAs, B10f, B20f or B30f convolution kernels, CTDIvol of 11.4 ± 6.6 mGy, DLP in the range 253.5/1228 mGy*cm. All images were sent to our picture archiving and communications system (PACS).

### Images analysis

All studies were reviewed by two radiologists, with 5- and 15-years’ experience in thoracic imaging. The entire volume of the primary tumour was visually segmented in both unenhanced and enhanced acquisitions employing a volumetric ROI (region of interest) using 3DSlicer software version 4.10.2 [25]. Textural features extraction was carried out through SlicerRadiomics tool. A total of 107 features of the PyRadiomics lists were selected, belonging to First Order, Shape-Based 3D, Gray Level Co-occurrence Matrix (GLCM), Gray Level Size Zone Matrix (GLSZM), Gray Level Run Length Matrix (GLRLM), Neighbouring Gray Tone Difference Matrix (NGTDM) and Gray Level Dependence Matrix (GLDM) classes [26].

### Statistical analysis

Nonparametric tests were performed to identify features that showed significant differences between the three classes of Ki67 or between the presence or absence of mediastinal lymph node metastases. This statistical analysis was performed separately on unenhanced and on contrast-enhanced CT scan databases. For the Ki67 class distinction, the Kruskal–Wallis test was used and the post hoc analysis was performed with the Dunn’s test, considering the Bonferroni correction. For the metastases grouping distinction, the Mann–Whitney test was employed. The significance threshold was set at *p* = 0.05. Statistical analysis was performed using SPSS (IBM SPSS Statistics for Windows, Version 27.0. Armonk, NY: IBM Corp).

## Results

**Fig. 2:**
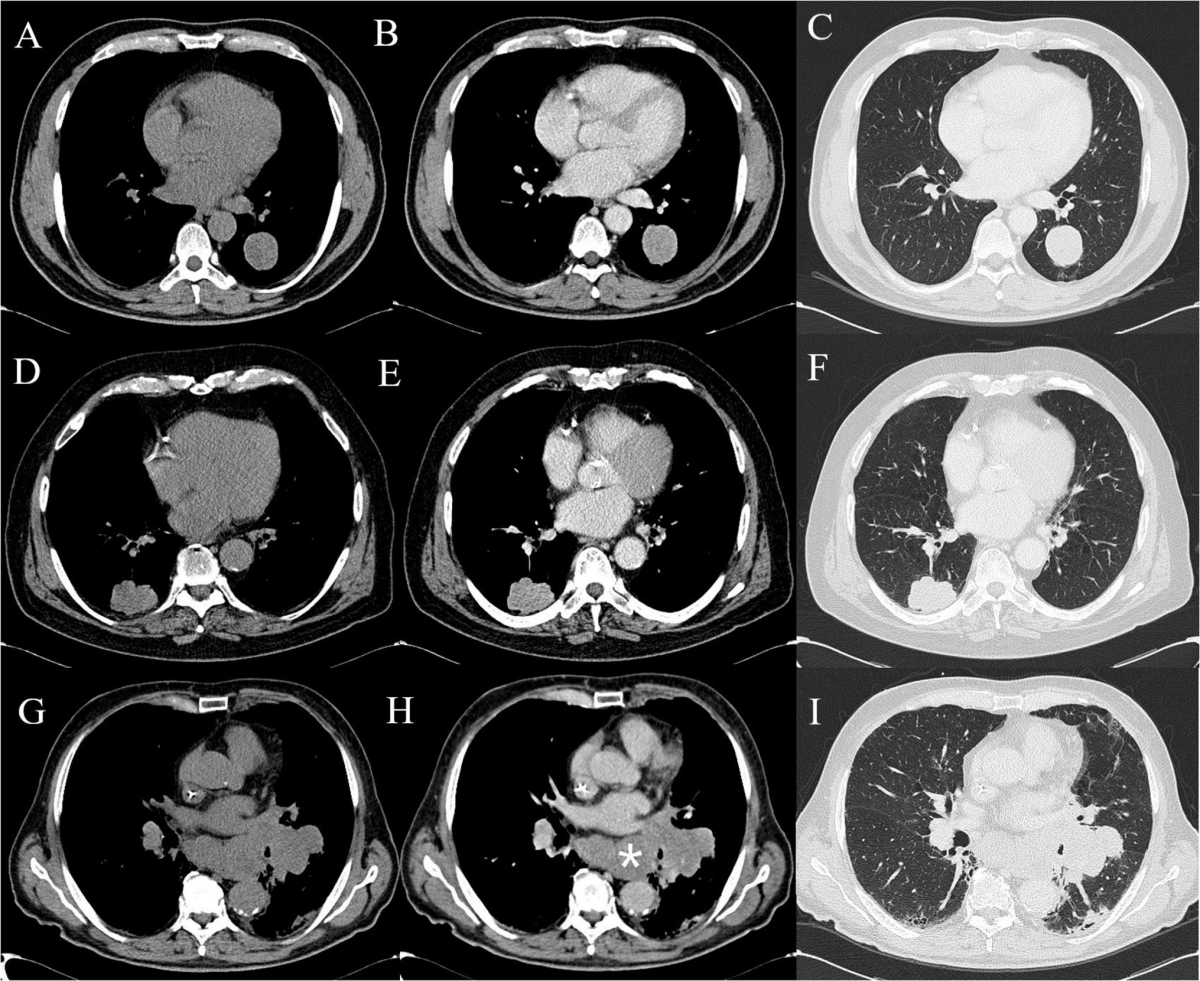
Computed tomography imaging in a 67-year-old male patient with a smooth-edged pulmonary lesion with histology of a typical carcinoid in the left lower lobe at baseline (**A**), with vivid enhancement in the venous phase (**B**) and lung parenchyma window (**C**). Large cell carcinoma in a 79-year-old male patient closely connected to the right posterior costal pleura; it shows polylobulated edges at baseline and lung parenchyma window (**D**, **F**) with contrast enhancement (**E**). Small cell carcinoma in a 76-year-old female patient: large left hilar lesion on direct examination (**F**), with inhomogeneous enhancement (**G**) and in parenchyma window (**H**). Notice the multiple confluent mediastinal lymphadenopathies (asterisk)

**Fig. 3:**
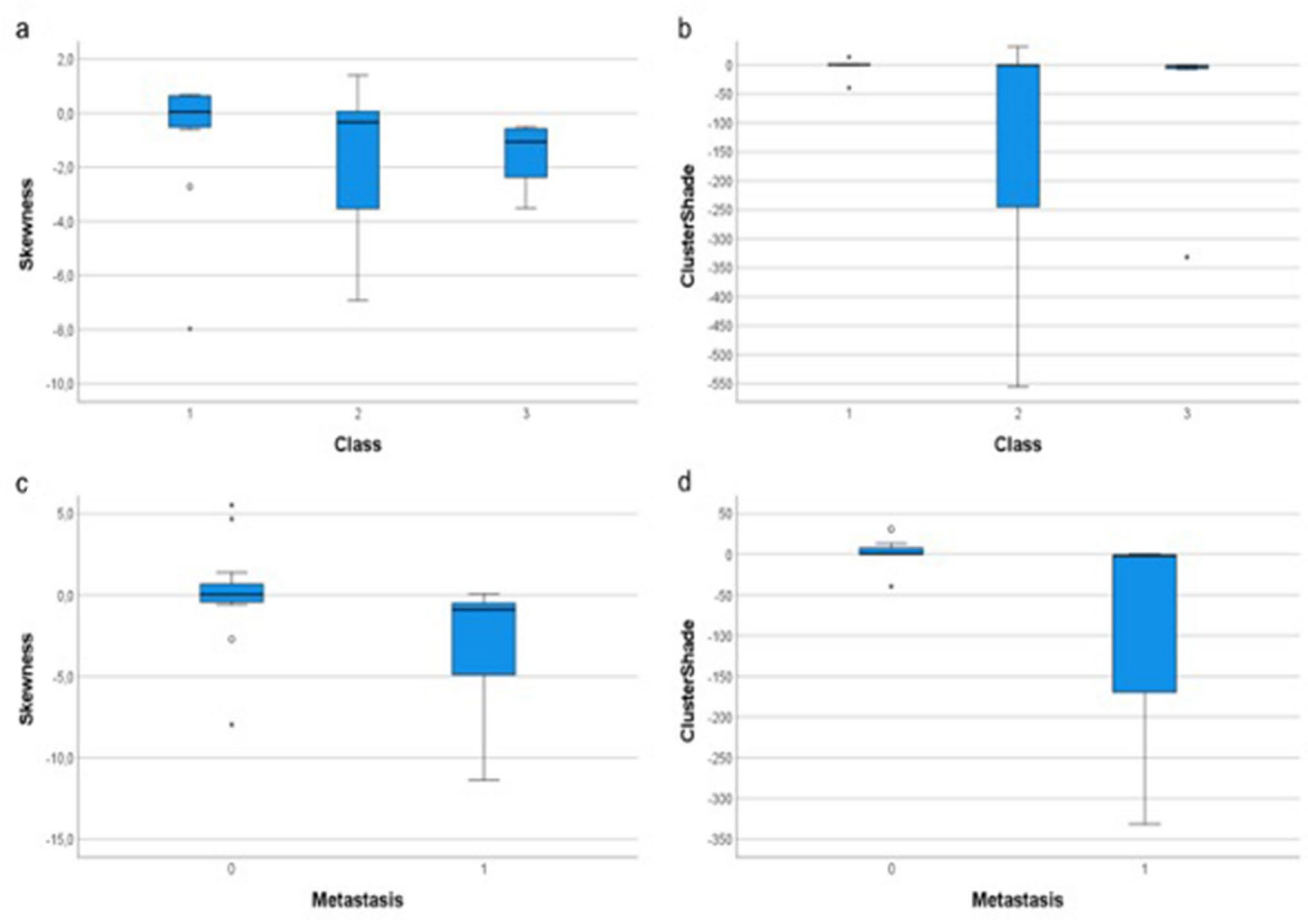
**a** Boxplot of Skewness feature vs Ki-67 classes; **b** boxplot of ClusterShade feature vs Ki-67 classes; **c** boxplot of Skewness feature vs absence (0) or presence (1) of metastases; **d** boxplot of ClusterShade feature vs absence (0) or presence (1) of metastasis

This retrospective observational study was approved by the Ethics Committee of our Institution (study protocol n:14776_oss). Finally, 27 patients were selected: 11 males and 16 females aged between 48 and 81 years old (average age of 70.4 years). Histopathological analysis revealed 21 cases of TCs (77.8%), 3 SCLCs (11.1%) and 3 LCNECs (11.1%). According to Ki-67 expression, patients were divided into Group 1 (13/27, 48.2%), Group 2 (7/27, 25.9%) and Group 3 (7/27, 25.9%). Mediastinal lymph nodal metastases were present in 12/27 (44.4%) at the first follow-up made by FDG-PET/CT. Eleven cases (52.4%) of TCs present as parenchymal mass (greater than 3 cm in diameter), all with regular margins and intense enhancement (Fig. 2). In contrast, the remaining 10 (47.6%) TCs were nodules with a diameter of less than 3 cm. Of these, 8/10 (80%) showed regular margins and intense enhancement. The presence of calcifications within the lesion was assessed more frequently in the larger TCs (being present in 9/11 masses and in 2/10 nodules). Broncho-stenosis associated phenomena were reported in 6/21 TCs (28.6%), mainly in those larger than 3 cm. In contrast, LCNECs and SCLCs showed variable sizes with irregular margins and predominantly inhomogeneous enhancement. Calcifications were found in only one case and broncho-stenosis was more frequent (4/6 cases, 66.7%). Typical carcinoids showed mean Ki67 values of 5.14, while both LCNECs and SCLCs of 80.0 [27]. Significant associations between textural features and Ki-67 classes and with the presence of lymph node metastases were detected only on contrast-enhanced CT images; no significant association was thus detected on unenhanced images. In Table 1, the features that showed significant differences among the Ki-67 classes after Kruskal–Wallis with Dunn’s post-hoc test are listed: median and 1° and 3° Quartile of each feature value in each group are also reported (Table 1). In Table 2, the features that showed significant differences among patients with and without detectable lymph node metastases after the Mann–Whitney test are listed: mean, standard deviation and range of each feature value in each group are also reported (Table 2). It is noteworthy that some features showed a marked difference in values between different classes of Ki-67; among these, Skewness and ClusterShade showed marked differences also between the groups of patients with and without lymph node metastases detected on follow-up CT examination, showing very low *p* values (*p* < 0.01). These results suggest that the values of these two features may be associated with the severity of the disease. Skewness and ClusterShade boxplots with the values that these features showed in the different groups are reported (Fig. 3).

**Table 1:** List of significant features of the nonparametric test between the 3 Ki-67 classes

Feature	Ki-67 class	Median	1°/3° Quartile	*p* Value	Dunn’s test
Maximum	1	174	144/333	0.010	1–2, 2–3
	2	228	196/259		
	3	134	129/151		
DependeceEntropy	1	5.76	5.53/5.90	0.024	2–3
	2	6.00	5.91/6.11		
	3	5.49	5.25/5.77		
ClusterShade	1	0.57	− 0.26/1.87	0.039	1–3
	2	− 0.37	− 244.98/0.38		
	3	− 1.94	− 6.14/− 1.61		
Correlation	1	0.36	0.33/0.59	0.048	
	2	0.51	0.37/0.57		
	3	0.24	0.12/0.33		
RootMeanSquared	1	98.8	81.1/129.7	0.050	
	2	101.6	88.6/108.2		
	3	67.6	59.3/82.3		
Skewness	1	0.054	− 0.52/0.64	0.050	
	2	− 0.33	− 3.53/0.065		
	3	− 1.05	− 2.36/− 0.58		

For each feature *p* value and pair that had passed Dunn’s test, if present, are reported. Two features close to significance (in round brackets) are also reported

**Table 2:** List of significant features for the Mann–Whitney test between absence (0) or presence (1) of metastases

Feature	Metastases	Median	1°/3° quartile	*p*-Value
ClusterShade	0	0.57	− 0.28/7.67	0.004
	1	− 2.05	− 170/− 0.91	
Skewness	0	0.054	− 0.42/0.66	0.006
	1	0.91	− 4.90/− 0.51	
LargeAreaLowGrayLevelEmphasis	0	435	331/730	0.032
	1	276	22.3/365	

For each feature are reported median and Tukey’s 1° and 3° quartile separately for the two groups and the *p* value

## Discussion

To our knowledge, this is the first study that used a radiomic approach to evaluate aggressiveness features of pulmonary NETs and several observations can be made from these results. The main result is that there are two features (Skewness and ClusterShade) that have significant differences between different Ki-67 classes and the presence or not of lymph nodal mediastinal metastases. Skewness is a first-order feature representing the asymmetry of the pixel values distribution from the mean value: if the value is zero, the distribution is symmetrical to the mean. The skewness would therefore be a representation of the internal structural inhomogeneity of the lesion, such as during necrotic-colliquative phenomena, certainly more frequent in the case of large or more aggressive masses. Our results show a clear stratification between patients with metastases (negative Skewness values) and without (Skewness around value 0). Moreover, grade 2 and 3 NETs have lower values of Skewness, showing that this feature could be useful in evaluating lesions with a high rate of cellular proliferation and aggressiveness [28]. ClusterShade is a second-order feature that gives a measure of the image non-uniformity. The results show a correlation with the presence of metastases and with Ki-67 values, affirming in fact that a greater tissue inhomogeneity is related to a greater aggressiveness of the disease [18]. From our results, these two features seem to be good candidates for assessing disease severity. This is supported also by the fact that these are correlated with each other and are first and second-order features, thus quite robust. Significant associations between textural features and Ki-67 classes and with the presence of metastases were detected only on contrast-enhanced CT images; no significant association was thus detected on unenhanced images. This is confirmed by the fact that CT examination without contrast medium does not provide much more information than anatomical-morphological ones, and at least one post-contrast phase is necessary, particularly in the staging of tumour pathology [29–31]. Moreover, in our results, the majority of patients have nodules or masses with high contrast enhancement, which is a typical characteristic of the spectrum of all neuroendocrine tumours, even those in the lung [21, 23, 24, 27]. This study has several limitations, first of all, the small number of selected cases. It has to be remembered that NET is a very rare lung neoplasm and that the selection of patients was accurately done to obtain a sample as homogeneous as possible in twelve years of data collection. Nevertheless, the analysis protocol is not the same, although only one CT scanner was used (slice thickness between 1 and 5 mm). In addition, there was a lack of normalisation of the results, a problem partially limited by the fact that only one CT scan was considered [32, 33]. Among future developments, it would be interesting to repeat the evaluation on a different dataset of patients (possibly also with different CT tomography and acquisition protocol), to confirm or not the association of the two features with disease severity [34, 35]. This is an initial evaluation and further studies would be needed to define a precise “radiomic signature” associated with NET severity.

To the best of our knowledge, no correlation has been reported in the literature between radiomics analysis of NETs of the lung and their stratification according to Ki-67 value and the presence or absence of metastases. Despite radiomics has not yet been translated into clinical practice, radiomics feature-based evaluation of primary pulmonary NETs has the potential for predicting occult metastases. CT texture analysis may be used as a valid tool for predicting the subtype of lung NET and its aggressiveness, thus aiding in accurate multidisciplinary decision-making.
